# How Aging and Oxidative Stress Influence the Cytopathic and Inflammatory Effects of SARS-CoV-2 Infection: The Role of Cellular Glutathione and Cysteine Metabolism

**DOI:** 10.3390/antiox11071366

**Published:** 2022-07-14

**Authors:** Francesco Galli, Giada Marcantonini, Daniela Giustarini, Maria Cristina Albertini, Anna Migni, Linda Zatini, Antimo Gioiello, Ranieri Rossi, Desirée Bartolini

**Affiliations:** 1 Department of Pharmaceutical Science, Nutrigenomics and Micronutrient Vitamins Lab and Anatomy Lab, University of Perugia, 06126 Perugia, Italy; giadamarcantonini94@gmail.com (G.M.); annamigni4@gmail.com (A.M.); lindazatini94@gmail.com (L.Z.); antimo.gioiello@unipg.it (A.G.); desiree.bartolini@unipg.it (D.B.); 2 Department of Biotechnology, Chemistry and Pharmacy, University of Siena, 53100 Siena, Italy; daniela.giustarini@unisi.it (D.G.); ranieri.rossi@unisi.it (R.R.); 3 Department of Biomolecular Sciences, University of Urbino Carlo Bo, 61029 Urbino, Italy; maria.albertini@uniurb.it

**Keywords:** SARS-CoV-2, inflammation, cellular redox, oxidative stress, thiols, aging, lung diseases, cytokines, glutathione

## Abstract

SARS-CoV-2 infection can cause a severe respiratory distress syndrome with inflammatory and thrombotic complications, the severity of which increases with patients’ age and presence of comorbidity. The reasons for an age-dependent increase in the risk of severe COVID-19 could be many. These include defects in the homeostatic processes that control the cellular redox and its pivotal role in sustaining the immuno-inflammatory response to the host and the protection against oxidative stress and tissue degeneration. Pathogens may take advantage of such age-dependent abnormalities. Alterations of the thiol redox balance in the lung tissue and lining fluids may influence the risk of infection, and the host capability to respond to pathogens and to avoid severe complications. SARS-CoV-2, likewise other viruses, such as HIV, influenza, and HSV, benefits in its replication cycle of pro-oxidant conditions that the same viral infection seems to induce in the host cell with mechanisms that remain poorly understood. We recently demonstrated that the pro-oxidant effects of SARS-CoV-2 infection are associated with changes in the cellular metabolism and transmembrane fluxes of Cys and GSH. These appear to be the consequence of an increased use of Cys in viral protein synthesis and to ER stress pathway activation that interfere with transcription factors, as Nrf2 and NFkB, important to coordinate the metabolism of GSH with other aspects of the stress response and with the pro-inflammatory effects of this virus in the host cell. This narrative review article describes these cellular and molecular aspects of SARS-CoV-2 infection, and the role that antivirals and cytoprotective agents such as N-acetyl cysteine may have to limit the cytopathic effects of this virus and to recover tissue homeostasis after infection.

## 1. Introduction

SARS-CoV-2 infection causes COVID-19. This can present with a respiratory distress syndrome and inflammatory and thrombotic complications, the severity of which increases with patients’ age and with the presence of comorbidity [1].

The age-dependent alterations of physiological functions that increase the risk of severe COVID-19 are little understood. These are expected to include defects in fundamental homeostatic mechanisms, such as those involved in the control of tissue redox being these essential to defining the individual susceptibility to oxidative stress, the efficacy of immune function and the modulation of inflammatory pathways [2].

In this respect, alterations of the levels and redox balance of cellular and extracellular thiol have been alleged to influence the risk of infections and severe complications in the elderly. The reduced to oxidized ratio of non-protein, or Low Molecular Weight (LMW), thiols and the levels of protein Cys thiolation in extracellular fluids could help to weight such risks. In fact, the redox balance of extracellular thiols is a reliable biological indicator of the age-dependent decline of the systemic redox [2,3,4,5] and its metabolic and functional impact on tissues, including the lung [6,7].

Such an age-dependent decline is even faster in patients affected by inflammatory and degenerative diseases, including those of the respiratory tract. In fact, independently from their origin, chronic and infectious pulmonary diseases can alter the redox of thiol species in both the blood plasma and extracellular fluids of the airways (reviewed in [7,8,9]); alterations of lung concentrations and detoxification function of cellular glutathione (GSH) have also been reported in these diseases (recently reviewed elsewhere in [2]).

Recent evidence in the literature suggests the hypothesis that viral infections may take advantage of an age-dependent defect in lung thiols to develop. In the case of extracellular thiols, increased levels of oxidation have been speculated to influence both the ACE-2 receptor-dependent mechanism of viral infection (reviewed in [2,10]), and the host capability to respond to the pathogen [11].

Moreover, a pro-oxidant environment in the host cell is proposed to sustain the replication cycle of SARS-CoV-2 and other viruses such as HIV, influenza, and HSV [9,12]. Rather, these viruses appear to actively promote such pro-oxidant conditions in the host cell and in the case of SARS-CoV-2 infection, we recently demonstrated that this occurs by the induction of specific defects in the transcriptional mechanisms that control the cellular metabolism and transmembrane fluxes of Cys and GSH as components of the adaptive stress response of the cell [8].

Transcriptional effects of this infectious disease also include a marked activation of inflammatory pathways and pro-inflammatory cytokine production in the host cell [13]. NFkB appears to play a role in this respect, and it is considered a pharmacological target of SARS-CoV-2 infection [14,15]; besides the transcriptional modulation of inflammatory genes, NFkB regulates several stress-associated genes, including those of the UPR and ER stress pathway, as well as cell cycle, autophagy, and apoptotic genes. At the same time, this transcription factor influences the metabolism and redox function of cellular GSH (reviewed in [16]). As a consequence, it is conceivable to speculate a role of NFkB activation in keeping together the inflammatory and cytopathic effects of SARS-CoV-2 infection with the alterations that this virus promotes on GSH metabolism and other thiols of the host cell [8].

These aspects are discussed in the present review article in relationship to the defective capability of the aging cell to deal with the redox challenges of SARS-CoV-2 infection, which may represent an underlying mechanism behind the age-dependent risk of developing oxidative stress and the inflammatory complications of severe COVID-19. Cytoprotective strategies that may limit the effects of SARS-CoV-2 infection on the cellular redox are also discussed in the context of antiviral therapy and ancillary treatments with Cys analogues such as N-acetyl cysteine (NAC).

## 2. The Role of Thiols in Maintaining the Redox Balance of Tissues

The nucleophilic reaction of LMW thiols with protein Cys residues involved in disulphide bonds produce thiol-disulphide exchange reactions important in the redox buffering and antioxidant protection of cellular proteins. Mixed disulphides (PS-SR) are also produced by the reaction between PSH or PSSG and LMW-SH. These reaction processes are essential to the functional modulation of redox-sensing and signalling proteins [17]. The PSSP reduction by an LMW-SH follows the reaction scheme:

2RSH + PS-SP←→ 2PSH + RS-SR

(RSH = LMW-SH; PSH = protein SH; PSSP = inter or intra-chain protein disulphides).

This type of reaction exemplifies the role that GSH may have in the reduction of accessible intramolecular PSSP to form GSSG as oxidation product. In this context, the thiolates of the involved species are the actual reacting forms and PS-SR are generated as reaction intermediates (in the case of GSH the mixed disulphide corresponds to the glutathionylation product of the corresponding protein Cys residues involved in the disulphide bond). The type of thiol, the pKa of the nucleophilic thiol and that of the leaving thiol groups, and the presence of entropic barriers and steric hindrance effects, are all aspects that influence the molecular dynamics and the kinetics and equilibrium constants of these reactions [18].

**Figure 1:**
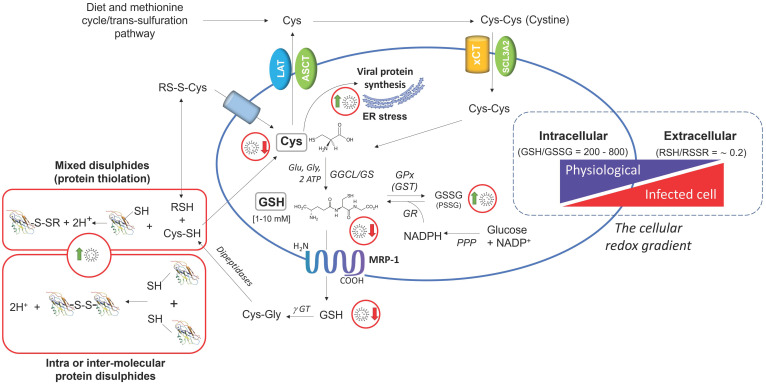
SARS-CoV-2 infection interferes with the bidirectional flux of cellular thiols (cystine-cysteine cycle) and glutathione metabolism. Cells actively control their redox state and the redox homeostasis of the extracellular environment, synthesizing the tripeptide glutathione and maintaining it in its reduced form (GSH). This latter process relies on the glycolytic activity of the Pentose Phosphate Shunt (PPP) that supports reducing equivalents (under the form of the pyridine coenzyme NADPH) to GSH-reductase (GR). The de novo biosynthesis process of glutathione depends on the cellular availability of Cys. Adequate Cys levels are provided through the diet and the trans-sulfuration pathway and reach the cells by means of the cystine-cysteine cycle. This cycle and the efflux mechanisms of cellular thiols maintain GSH, Cys, and the other LMW thiols available in the extracellular milieu for the reaction with protein thiols (left insert) and for the intercellular metabolism of GSH. At the same time, this cycle participates in the homeostatic control of the redox gradient existing between cells and extracellular fluids of tissues (right insert). The viral replication process implies an increased incorporation of cellular Cys in the viral proteins, thus reducing its availability for the biosynthesis of GSH and the redistribution of cellular thiols to the extracellular compartment. Aging conspires with SARS-CoV-2 infection to promote these changes in the metabolism of thiols, which may increase the risk of degenerative complications and severe COVID-19.

Cysteine/cystine (Cys/CySS), cysteinilglycine/cystinilglycine (CysCgly/CysGlySS), and glutathione/glutathione disulphide (GSH/GSSG) are the main redox-active LMW thiol/disulphide couples of extracellular fluids; these present an average molar ratio of 0.2 in human plasma in the presence of total concentrations of Cys and GSH that are of approx. 200 μM and <10 μM, respectively [2] (Figure 1).

At the cellular level, GSH is the main LMW-SH (from 1 to 10 mM in the different types of cells), and the GSH/GSSG ratio ranges from 200 to 800 [16] (Figure 1). Cys and other LMW-SH are in the micromolar range in the cells, but also these thiols have the reduced form as predominating on the oxidized. Therefore, under homeostatic conditions, the intracellular environment presents as markedly reducing whereas the extracellular as oxidizing (Figure 1). Such a redox asymmetry, or gradient, is ensured by the combination of two main aspects: (1) the active transport of Cys into the cell; and (2) its utilization to synthesize GSH that is maintained reduced by the activity of a NADPH-dependent GSH-reductase (GR). The first depends on a series of transporters that ensure the bidirectional transport of LMW-SH thiols throughout the plasmalemma (Figure 1), with Cys and Cyss influx that prevail on efflux thus ensuring the Cys required for the biosynthesis of GSH and cellular proteins. Efflux mechanisms redistribute cellular Cys, GSH, and other LMW-SH to other cells, thus ensuring their local and systemic availability [2]. The second aspect relies on the glycolytic activity of the pentose phosphate pathway (PPP) to produce the reduced form of nicotinamide adenine dinucleotide phosphate (NADPH), the coenzyme of oxidoreductases with a key role in cellular LMW-SH and protein Cys redox buffering. These include GR that catalyses the reduction of one molecule of oxidized glutathione (GSSG) to two molecules of GSH using NADPH as reducing cofactor:

GSSG + 2 NADPH → 2 GSH + 2 NADP^+^ + 2H^+^

(GSSG = oxidized glutathione; GSH = reduced glutathione; NADP = Nicotinamide adenine dinucleotide phosphate).

GSH and other reduced cellular LMW-SH formed through the activity of NADPH-dependent oxidoreductases, such as GR and thioredoxin reductase (TrxRD), are used by the same TrxRD and other downstream oxidoreductases, such as glutaredoxins and sulfiredoxins, which constitute the redox buffering platform of cellular proteins [19]. Data obtained in cell culture model-systems, such as HEK and HeLa cells, indicate that this platform maintains the large majority of protein thiols in the reduced state (more than 90% of total protein thiols), which may lead us to speculate a redox buffering role for this pool of cellular thiols comparable or even superior to that of GSH [20]. Thus, PSSP and PSSG forms account for a minor fraction of the cellular pool of protein thiols (5–10% and <0.1%, respectively). However, the levels of these oxidized forms, and particularly of PSSG, increase by at least one order of magnitude during the exposure to thiol-specific oxidants such as diamide [20] or seleno-organic agents [21]. Likewise, PSSG are markedly induced by the manipulation of genes responsible for the transcriptional control of thiol oxidoreductases and PPP activity of the cell [21].

**Figure 2:**
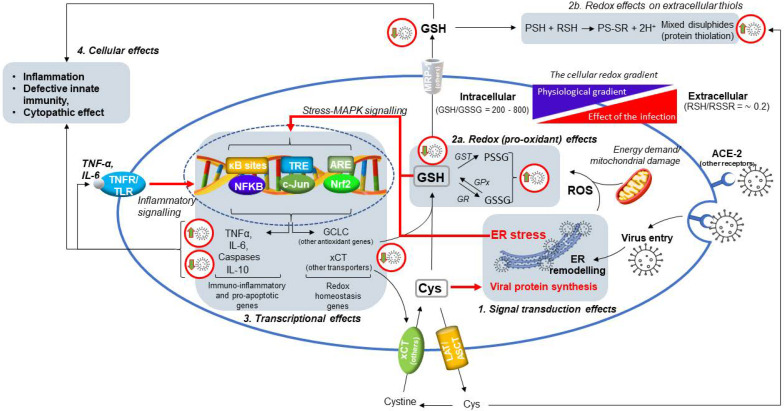
Effects of SARS-CoV-2 infection on the Cys/GSH system and its interplay with signal transduction and transcriptional mechanisms that sustain the pro-inflammatory and cytopathic outcomes of this infection in the host cell. Key steps in the molecular and cellular effects that link these events together are identified from experimental data obtained in Vero E6 epithelial cells [8,13]: 1. signal transduction effects are produced post-infection by the induction of ER stress that follows the remodelling of this subcellular compartment by the abnormal synthesis and assembly of viral particles; 2. redox abnormalities are induced by the increased incorporation of Cys into viral proteins that inhibits the synthesis of cellular GSH (2a), and by the increased flux of cellular ROS sustained in the different cellular organelles by the viral replication, as ER and mitochondria. These alterations include effects on the extracellular redox by the reduced efficacy of redox buffering systems that rely on the efflux of GSH and other cellular thiols (2b); 3. transcriptional effects are induced by the effect of ER stress signalling and a defective metabolism of cellular GSH, which modulate the activity of transcriptional proteins, such as NFkB and Nrf2, during the viral replication cycle; 4. cellular effects develop as a consequence of these events. In fact, GSH metabolism inhibition, ER stress signalling and the activation of inflammatory genes may converge in the receptor-dependent and protein kinase-mediated activation of inflammatory and death pathways of the host cell, ultimately sustaining the cytopathic effect of the viral infection. These changes and the inhibition of GSH metabolism may also help the virus to evade innate immunity mechanism of the host cell. Red connectors highlight the main effects of SARS-CoV-2 infection on cellular pathways.

These genes include Nrf2 (Figure 2), one of the most important transcription factors involved in the transient response to cellular stressors [22,23], and especially to reactive oxygen species (ROS) produced for instance during cellular damage induced by different causes and in the inflammatory response, or during the exposure to xenobiotics with electrophilic properties and alkylating agents (thiol-specific oxidants) [21]. Under normal (unstressed) conditions, Nrf2 is bound by disulfide bridges to Keap1 which facilitates molecular complex ubiquitination and proteasomal degradation. During the exposure to cellular pro-oxidants and oxidative stress, stress-sensing cysteine residues engaged in the intermolecular bonds of Keap1-Nrf2 complex are oxidized, leading to Nrf2 dissociation and translocation into the nucleus to bind specific consensus sequences, identified as antioxidant or electrophile responsive elements (ARE/ERE), in the promoter region of stress response genes, including those that maintain the glycolytic flux of PPP and the oxidoreductases involved in the redox buffering of cellular thiols. Nrf2/Keap1 complex activation can also occur through the activity of specific protein kinases that keep Nrf2 activity coordinated with other cellular functions and stress response nodes to ensuring adaptive homeostasis towards different types of transient stimuli and variations of the redox tone. The activity and these interactions of Nrf2 are also important in the redox reprogramming of cancer cells to control cell death and autophagy pathways, and to support drug resistance mechanisms [24,25].

NFkB and AP-1 are other redox-sensitive transcription factors important for the response to different cellular stressors (Figure 2); the characteristics of NFkB and its role in the cellular effects of SARS-CoV-2 infection are described in Section 6, whereas AP-1 is a protein complex comprising c-Jun and c-Fos components, which play a key role in the transcriptional control of cellular GSH (recently reviewed in [26]).

These transcriptional proteins, namely Nrf2/NFkB/AP-1, present one or more consensus sequences in the promoter region of the majority of antioxidant and detoxification genes, including the genes that control both the constitutive and inducible levels of GSH. In fact, their transcriptional activity participates in regulating the expression of membrane transporters involved in the cysteine-cystine cycle and that of γ-glutamyl-cysteine lyase (GCL), which are the critical steps of GSH biosynthesis (introduced earlier in this section and described in more detail elsewhere in [16]). These steps control the cellular availability of Cys and its utilization to synthesize the intermediate γ-glutamyl-cysteine for the condensation with the amino acid glycine through the activity of GSH-synthetase in the second phase of GSH biosynthesis. The same transcriptional players coordinate the expression of genes that control the glycolytic flux of the cell for the production of energy (2 mol of ATP are needed to synthesize 1 mol of GSH) and reducing equivalents under the form of the GR cofactor NADPH produced in the PPP (Figure 1).

In contraposition to intracellular conditions, the extracellular environment lacks efficient redox homeostasis systems [27]. Rather, this compartment is characterized by the activity of oxidoreductases that sustain thiol-disulphide exchange processes [28]. Therefore, a more oxidized environment can increase the probability that solvent-exposed Cys residues of proteins present in biological fluids and on the cellular surface may react with other proteins or LMW thiols to form intra or intermolecular disulphides (Figure 1). For example, in the case of albumin, the most abundant plasma protein, only one solvent exposed Cys residue is present in position 34, and approximately half of the protein molecules present this highly reactive Cys residue under the PSSR form by the reaction of with LMM-SH.

It is worth noting that the efficacy of both the cellular and extracellular processes described in this section show an age-related decline (introduced in Section 1), thus increasing the risk of defects in adaptive homeostasis mechanisms of tissues and of oxidative stress, which are main pathogenic events in both the chronic and communicable diseases of the elderly. Mechanistically, such a decline can involve Nrf2 and the other aforementioned stress response pathways with a role in GSH metabolism regulation and in the adaptive homeostasis processes of the cell [22,29,30]. These changes affect the redox balance of extracellular thiols, with the percentage of extracellular PSH oxidized to PSSR (namely, the Protein Thiolation Index) that linearly increases during aging and with the levels of oxidative stress [31,32]. Such age-dependent redox disturbances and their development at the two sides of the cellular membrane, are discussed in the next sections in the context of SARS-CoV-2 infection and its cytopathic and pro-inflammatory outcomes in the host.

## 3. Age-Dependent Changes of Lung Thiols and the Role of GSH in Lung Diseases

Thiols represent the first line of defence against harmful pro-oxidants and the main redox buffering system of cells and tissues. The lung is one of the organs richest in thiols after the liver, kidney, and testis, and it is considered a site of storage for the cellular thiol GSH [7,33]. In addition, GSH is released in the epithelial lining fluids of the lung to control their physical and biological properties including fluidity, protection of the cell surface, and response to pathogens [34,35,36].

Studies in human bronchial epithelial cells [30] and in animal models [37] indicate that the age-related reduction in the cellular levels of GSH, described in the Section 2, can harm the cellular components of the lung, suggesting the existence of a progressive and age-dependent decline in the redox buffering properties of this tissue. Lung comorbidity may play a role in sustaining such a redox decline; for example, in the bleomycin model of pulmonary fibrosis, GSH/GSSG and Cys/Cyss decreased in plasma during the inflammatory and fibrotic phase of lung injury [38]. Alterations of these redox balances are also observed in cases of increased free radical generation and oxidative stress [37], which are aging accelerators and key pathogenic factors of both acute and chronic lung diseases, including pulmonary infectious diseases such as COVID-19 [11,36,39].

Likewise to what has been reported in animal models, the levels and protection function of lung GSH decrease with age also in humans, and even more in the presence of comorbidity and an unhealthy lifestyle, including smoking [3,33,40,41], which are all aspects associated with increased risk of severe COVID-19.

In addition, dietary habits and nutritional aspects have to be taken into account. An adequate intake and systemic availability of Cys [41,42], is important to sustaining the cellular biosynthesis of GSH and its distribution to all tissues, including the lung. Aging and age-associated comorbidity can lead to malnutrition and insufficient intake of sulphur-containing amino acids through different mechanisms, which may interfere with redox homeostasis mechanisms of tissues, include lack of appetite and dysphagia, changes in lifestyle and psychological aspects, and metabolic defects [41,43,44].

Therefore, to sum-up, aging and chronic diseases sustain oxidative stress and interfere with the physiological processes that control the redox homeostasis of the lung and other tissues and organs [3,4,44]. These latter include the transcriptional and metabolic processes associated with adaptive homeostasis mechanisms discussed earlier in Section 2, and these defects are exacerbated in case of premature aging and oxidative stress induced by chronic and degenerative ailments. For example, in chronic kidney disease, defects in the cellular levels of reduced pyridine nucleotides and GSH metabolism are associated with poor capacity to prevent oxidative damages of blood cells and to restore the extracellular redox [41,45,46,47,48]; even more evident is the case of chronic respiratory syndromes that induce defects in the levels of lung GSH and in the redox buffering of protein Cys (reviewed in [7,34,35,36,49] and references therein). Worthy of note is the fact that such prototypical examples of chronic ailments, and others associated with oxidative stress, are included in the comorbidity that increases the risk of severe COVID-19 [6,50].

The role of lung GSH in reducing the risk of infections is demonstrated by the evidence that defects in its biosynthesis and function contribute to an impaired immunity in the diseased host [51]. Accordingly, changes in the blood levels and detoxification function of lung GSH have been associated with a higher severity of clinical manifestations in COVID-19 [52,53,54].

Besides immune-inflammatory competences, lung GSH is also important to control fibrotic pathways of the tissue during chronic exposure to lung toxicities and cytopathic agents, and the induction of inflammatory and fibrotic genes is reported to deplete the lung tissue of GSH, interfering with its cellular biosynthesis [55].

**Figure 3:**
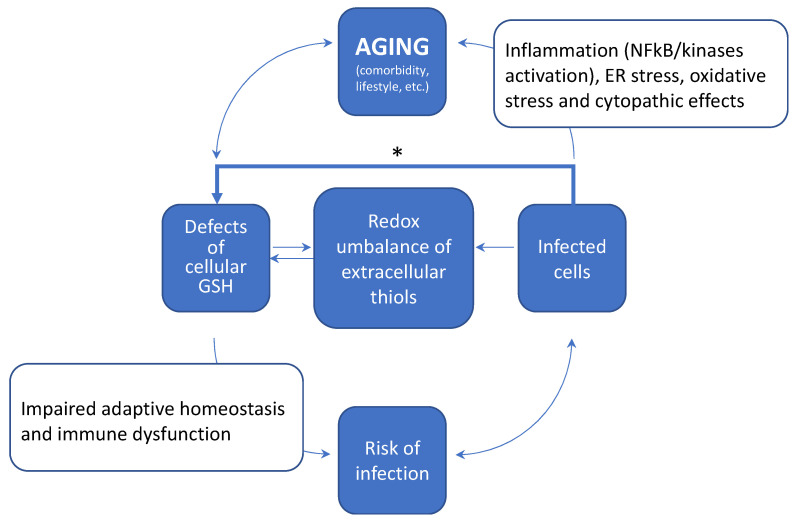
Age-dependent changes of tissue redox and risk of severe COVID-19. Aging promotes a progressive decline of physiological processes that modulate the tissue redox, also identified with the all-encompassing definition of “adaptive homeostasis” [29]. These processes include the homeostatic control of the levels and redox buffering properties of cellular thiols. Defects in these fundamental components, increase the risk of oxidative stress and immune dysfunction in the diseased host, ultimately reiterating the risk of SARS-CoV-2 infection and complications. In the infected cell, a decreased efficiency of the cellular metabolism and redox function of GSH is observed [8] (marked with the symbol * and discussed in Section 4) and this interferes with the redox balance of extracellular thiols, further sustaining the risk of infections and the age-related defect of thiol metabolism in the infected tissue and in the whole organism. SARS-CoV-2 infection also sustains stress kinase signalling and NFkB activation, thus stimulating the production of inflammatory cytokines in the host cell [13,58]. These pro-inflammatory events observed at the cellular level conspire with the redox defects of tissues to increase the risk of immune dysfunction and inflammatory complications that characterize the comorbidity of severe COVID-19.

Therefore, the whole picture indicates that aging promotes a progressive decline in the cellular metabolism and levels of GSH, thus hampering the physiological control of the tissue redox and increasing the risk of oxidative stress and immune dysfunction in the diseased host. A simplified scheme of how these age-dependent changes may increase the risk of infections and severe comorbidity in COVID-19 is depicted in Figure 3. Furthermore, pathogens and especially viral infections, have been reported to interfere with the cellular metabolism of thiols [9,12], and these include SARS-CoV-2 infection [8] (discussed in Section 4) that may contribute to the worsening of the age-dependent decline in the redox balance of extracellular thiols. Key mechanistic aspects of these alterations in the redox homeostasis of tissues are a defective metabolism and redox balance of cellular GSH, and their coupling with a modified cystine-cysteine cycle (Figure 1). These defects can induce a pro-oxidant environment both in the infected cell and extracellular fluids (Section 5) that may favour viral replication and spreading throughout the tissues. SARS-CoV-2 infection also induces stress kinase signalling and NFkB activation, thus sustaining inflammatory cytokine production and cell death mechanism in the host cell (Section 6; Figure 2). These responses can conspire with the redox defects of the aging tissue to increase the risk of immune dysfunction and inflammatory comorbidity that characterize the severe COVID-19. Moreover, NFkB transcriptional activity participates to control GSH levels in experimental models of cellular stress [56] and lung inflammation [57], which may offer a mechanistic link between the inflammatory and thiol-depleting effects of SARS-CoV-2 infection observed at the cellular level [8].

These aspects provide a solid background to hypothesize that changes in cellular thiols, and especially GSH, may play a role of in the age-associated pathophysiology of lung complications and severe COVID-19 (Figure 3), that is worth investigating at the clinical level.

## 4. SARS-CoV-2 Infection Impairs the Metabolism and Redox Function of Cellular GSH

SARS-CoV-2, likewise other viral infections, such as HIV, influenza, and HSV [9,12], promotes an oxidized environment in the host cell inducing depletion of cellular thiols and particularly GSH [8]. As introduced earlier, this tripeptide is one of the main antioxidants and detoxification metabolites (phase 2 drug metabolism substrate) of cellular systems, with a key role in maintaining all main physiological functions of epithelia and immune cells of the lung and other tissues [16]. The cellular metabolism of GSH is regulated by metabolic, nutritional, and transcriptional processes that govern the adaptive homeostasis of tissues. Because these processes show a characteristic age-related decline (discussed in Section 3), in the lung tissue of the elderly, this may synergize with the thiol-depleting effects of SARS-CoV-2 infection to increase the risk of severe lung damage, and respiratory and inflammatory complications.

However, the specific mechanisms by which this and other viral infections interferes with the metabolism of cellular GSH and other thiols, remain poorly understood. Recent studies carried out in our laboratories have provided insights on these cellular mechanisms of SARS-CoV-2 infection in Vero E6 kidney epithelial cells [8] (described in detail in Figure 2). Once infected, these cells show a marked reduction of cellular thiols with both the uptake of Cys and GSH biosynthesis that significantly decrease. The efflux of cellular thiols in the extracellular milieu is also reduced, which may support the hypothesis that SARS-CoV-2 infection promotes a pro-oxidant environment in the infected tissue, interfering with the cystine-cysteine cycle of the cell and thus with the redox homeostasis mechanisms of extracellular thiols shown in Figure 1 and further discussed in Section 5.

In addition, increased levels of GSSG and protein glutathionylation (PSSG) were observed in SARS-CoV-2 infected cells that showed a characteristic glutathionylation pattern of cytosolic proteins compared to uninfected cells [8]. This specific finding demonstrated at the same time that the cellular proteome is affected by the pro-oxidant effects of the viral infection and that specific protein targets are involved, the role of which in cellular damage and cytopathic effect (CPE) of this virus is worth investigating.

Mechanistic aspects behind these effects of SARS-CoV-2 infection include changes in the expression and transcriptional activity of Nrf2 [8], which participates in the modulation of genes associated with the de novo biosynthesis and NADPH-dependent reduction of GSH (see Section 2).

**Figure 4:**
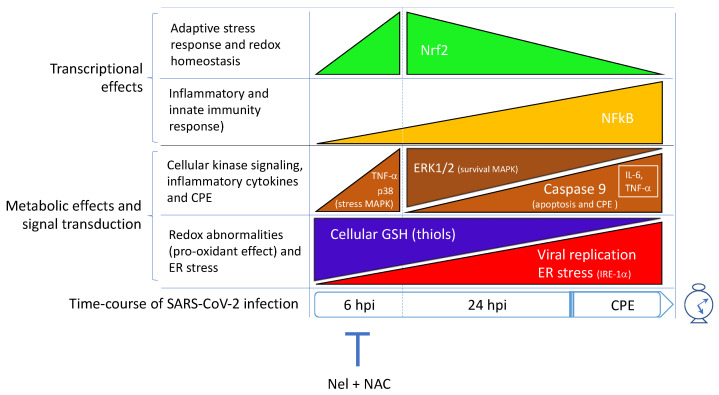
Signal transduction, transcriptional and metabolic effects of SARS-CoV-2 infection in Vero E6 cells. The time-course of the main effects shown in the chart include the modulation of stress response and apoptotic pathways, inflammatory genes and innate immunity of the host cell that were identified according to [8,13]. The reported time-points were 6 h post infection (hpi) and 24 hpi; these indicate the earliest and late phase of the viral replication process in which the cytopathic effect (CPE) of the virus develops, respectively. Nelfinavir (Nel) and N-acetyl cysteine (NAC) reverse the reprogramming effect of SARS-CoV-2 infection on the transcriptional and metabolic parameters of the host cell, acting early during the viral replication process to prevent ER reprogramming and Cys consumption for viral protein synthesis (shown in Figure 1 and Figure 2).

Furthermore, in these studies, the treatment with antiviral agents, such as Nelfinavir (Nel) and other protease inhibitors, and the nucleotide analogue Remdesivir (Rem), allowed a first exploration of the role that the different phases of viral replication may have on this transcriptional protein and its downstream genes (Figure 4). The findings in our studies demonstrated that the infection stimulates Nrf2 activity early in the cell infection program to satisfy the metabolic requirements of its replication cycle, reducing at the same time the efficacy of the Nrf2-dependent transcriptional mechanisms that sustain the synthesis of GSH as a part of the adaptive response of the host cell to the infectious agent. In fact, the infection itself produced a transient low-grade Nrf2 activation (observed at 6 hpi, but not at 24 hpi), that was associated with the same levels of induction of GSH biosynthesis genes, such as the enzyme subunit GCLC, and the membrane transporter MRP-1 that promotes thiol efflux and Cys redistribution among the cells through the Cys-Cyss cycle (Figure 1) [21]. Because of these effects, the viral replication process, at least initially, sustains Cys uptake to support viral replication but, in the end, the abnormal growth of viral protein synthesises results in cellular thiol depletion and activation of oxidative stress and death pathways (Figure 2); in this context, an efficient and timing block of viral replication by the antivirals, besides reducing the CPE of the virus, produced a marked Nrf2 activation response to the infection, almost completely restoring the levels of GSH and other cellular thiols [8]. This effect was obtained in the case of Nel, but not Rem, which was much less effective in reducing the viral replication in the Vero E6 cell model. Moreover, Rem induced Nrf2 activation in the late phase of the virus replication cycle, an effect that was incompatible with a significant restoration of cellular GSH.

These findings indicated that delaying the Nrf2 activation response could be a viral mechanism to reprogram the homeostatic mechanisms in order to keep the conditionally essential amino acid Cys available for the synthesis of viral proteins. In fact, a preferential incorporation of cellular Cys in the viral proteins rather than in GSH and cellular proteins is observed as a common mechanism also in other types of viral infections that, in turn, may hardly impact on the redox homeostasis of protein Cys residues in the host cell [12]. Other mechanisms for the GSH depletion effect of SARS-CoV-2 may include a cellular leakage of this tripeptide during the virus exocytosis process.

Besides an increased demand for Cys for the synthesis of viral proteins, the reasons for this and other viral infections to induce a defective metabolism of cellular GSH could include a mechanism for the virus to evade the cellular defences and the immune response that depends on the availability of this tripeptide and its redox-buffering effects [59,60,61].

However, regardless of the causal mechanism, a defective metabolism of cellular GSH causes cellular damage and the CPE of the virus by death programs activation in the host cell [21,62] (Figure 2). The glutathione system and its redox buffering effects on cellular protein Cys, have a prominent position in the pecking order of homeostatic responses that control the balance between cell survival and death pathways [63]. These include the unfolded protein response (UPR) and endoplasmic reticulum (ER) stress response, which are key players of SARS-CoV-2 CPE [13] (Figure 2). This is a characteristic response to the replicative process of different members of the Coronavidae family that characteristically stimulate UPR/ER stress, leading to autophagy or death programs activation in the host cell [64,65].

Under these conditions, the formation of mixed disulphides on cellular proteins during SARS-CoV-2 infection (mainly occurring by glutathionylation), can sustain CPE promoting protein damage and ER stress [9,13]. Important enough is that GSH depletion due to increased efflux of the tripeptide or reduced biosynthesis, is a signal for the cells that may conspire with ER stress signalling to activate death programs [66,67] (Figure 2).

These aspects may lead us to hypothesize that SARS-CoV-2 infection actively interferes with the metabolism of GSH in the host cell and with the role of this tripeptide in the homeostatic control of the cellular redox. This ultimately produces an optimal environment for the virus to synthesize its components during the replication process, also eluding the cellular defence systems.

According with this pro-oxidant role of SARS-CoV-2, the oxidation of cellular GSH to the corresponding disulphide GSSG was increased in infected Vero E6 cells [8], and an important finding in our studies was the ability of the Cys analogue NAC to reduce the levels of glutathione oxidation while the synthesis and efflux of cellular GSH were induced, thus increasing the redox buffering capacity of both the cellular and extracellular environment. Considering these cellular responses to such Cys analogue, other authors demonstrated that the pro-oxidant effect of the influenza virus in the host cell was reduced by the treatment with GSH analogues in in vitro models and in vivo old and lethally-infected mice. In addition, promising results on these agents have also been reported in other experimental models of viral infections [60,68,69]. Furthermore, treatments with Cys analogues, such as NAC, have successfully been utilized to replenish blood GSH and to improve immune and metabolic functions in HIV-infected patients as well as in many chronic respiratory diseases (reviewed in [2]).

## 5. How Aging and a Defective Cellular Redox May Conspire to Increase the Risk of Infection and the Development of Severe COVID-19

As the aging process and the effects of comorbidity impair the metabolism of tissue over a certain threshold level, the redox balance of extracellular thiols begins to fail, and oxidized forms become predominant over the reduced ones [2]. As introduced earlier in Section 2, this is the consequence of a defective capability of the cells to produce and deliver enough reducing equivalents to the cellular GSH and other cellular thiols, through the NADPH-generating function of PPP and the activity of cellular oxidoreductases. Such reducing power is available to control the extracellular redox by the efflux of GSH, Cys, and other reduced forms of cellular thiols that work as a Cys/H^(e−)^ shuttle system important for the redox homeostasis of tissues. This thiol system also benefits of the redox crosstalk with other redox buffering systems including endogenous antioxidants and micronutrients, such as ascorbic acid [70], that depend on the activity of specific cellular oxidoreductases.

All the metabolic aspects that control the levels and redox of cellular GSH, including its efflux together with other cellular thiols, are inducible processes (Figure 2 and Figure 4). These respond to a series of cellular stimuli, such as the exposure to xenobiotics, metalloids and heavy metals with electrophilic properties [21,71], and infectious agents such as SARS-CoV-2 [8]. However, the induction effect of SARS-CoV-2 infection on GSH metabolism and efflux appears to be limited to the earliest phase of the infection, ultimately promoting a depletion effect on cellular GSH that frustrates the cellular defence and the adaptive response to the infection (Section 4).

This also interferes with the capability of cellular GSH to sustain the redox buffering of extracellular thiols (Figure 1). A decline in the redox balance of extracellular thiols is proposed to influence the host response to the pathogens in different ways. First, immune cells depend on the availability of Cys to maintain their cellular functions, including the host response to antigens [52,72] and inflammasome activation in macrophages [72]. On the other hand, an increased efflux of GSH during cellular damage and oxidative stress can sustain the activation of cell senescence and death programs in immune cells and other cell types including epithelial cells [66,67].

Second, but not less important, the redox status of critical Cys residues on the extracellular domains of membrane proteins can be affected by the redox status and thiolation activity of extracellular LMW thiols (reviewed in [2]) that are important for different cellular functions, including membrane binding and cellular entry of viral particles [73,74]. The tropism of Coronaviruses is dependent on different mechanisms including the ability of the spike glycoprotein (S) to bind to ACE2 cell surface receptor. The receptor binds with high affinity the receptor binding domain (RBD) present on the spike protein, and subsequently the S protein is cleaved by the transmembrane protease serine 2 [75,76]. Biochemical studies have highlighted how the higher infective potential of SARS-CoV-2 compared to SARS-CoV lies in the fact that its spike protein has 20-fold higher affinity for human ACE2, with a Kd of 15 nM. Moreover, the infectivity of different SARS-CoV-2 strains correlates with the energy of RBDs and ACE2 receptor interaction [77,78]. In addition, the number of available ACE2 receptors is another factor that influence the virus replicative ability. The receptors are present in lung cells, but are rarely expressed in immune cells, where other receptors, such as CD147 and CD26 may have a role in mediating virus entry [79,80]. Worthy of note, ACE2 expression in most tissues declines with the host’s age; as a consequence, it is reasonable to hypothesize that other age-dependent factors have to intervene to eventually explain the increased risk of infection and severe disease with the age of the host [81]. Among these, defects in the adaptive homeostasis of tissues are worth considering [29]; aging is associated with a progressive decline of homeostatic systems that control the redox balance of cells and tissue, including lung epithelial cells [30], which may explain an increased susceptibility to develop pulmonary diseases in the elderly [7]. These alterations interfere also with tissue regeneration processes increasing the risk for viral infections.

Protein structure data and molecular dynamics studies have indicated that the disulphide-thiol balance of proteins at the viral surface may influence the binding of the spike protein to the ACE2 receptor in the host cell [10,82]. In more detail, in these studies it is suggested that a pro-oxidant environment may favour the virus-host cell interaction. In fact, the cleavage of the C344-C361 disulphide bridge present on the ACE2 receptor was hypothesized to cause conformational changes of the two α helices that bind with the spike RBD. Accordingly, the reduction of C480-C488 disulphide bridge on the spike protein binding domain may modify the β-sheet loop of the domain [10]. These molecular dynamics data can lead us to postulate that the binding affinity between the spike protein and ACE2 receptor grows with the presence of increased levels of oxidation in the extracellular LMW-thiols, the probability of which linearly rises with the patient’s age [2].

## 6. SARS-CoV-2 Infection Triggers NF-κB Activation and Inflammatory Cytokine Expression in the Host Cell

A sustained NF-κB signalling has been observed in SARS-CoV-2-infected human airway epithelial cells [58] as well as in Vero-E6 epithelial cells [13]. NF-κB is a ubiquitous transcription factor involved in the modulation of inflammatory genes and many other genes important in the host response and adaptive homeostasis [15,83]. NF-κB over-activation in the infected cell may explain the abnormal expression of inflammatory genes [8], including the cytokines TNF-α and IL-6, and the decreased expression of IL-10 that participates to limit the immune response to pathogens, thereby preventing damage to the host [84] (Figure 2). Such pro-inflammatory response was associated with the development of a pro-oxidant environment in the host cell that may combine with viral replication to induce cellular damage and UPR/ER stress pathway activation (described in Section 4 and in [13]) as main instigators of the CPE in SARS-CoV-2 infection (Figure 2).

Detailed characterization of these events in Vero-E6 cells demonstrated that cellular MAPKs are also modulated during the infection in a functional crosstalk with NF-κB and the UPR/ER stress pathway, as indicated by the induction of the UPR-dependent protein IRE-1α. An uncontrolled activation of ER stress pathway is a main underlying event in the CPE of this and other viral infections and ER stress may sustain NFkB activation also in other phases of the infectious cycle, thus keeping the transcription of pro-inflammatory and stress response genes activated in a signal transduction and transcriptional looping that ends in CPE execution (Figure 2). In fact, IRE-1α induction and dimerization on the ER membrane can sustain NF-κB stimulation by TRAF2 kinase activation and consequent IKK-mediated phosphorylation and proteolytic removal of the NF-κB inhibitor IKBα [85]. Considering the pro-oxidant and pro-inflammatory effects of SARS-CoV-2 infection, it is worth noting that this TRAF2-dependent pathway can be stimulated through cytokine receptors that can sustain NF-κB transcriptional effects via JNK activation (Figure 2). These kinases and the same NF-κB/IKK system are redox-sensitive signal transduction hubs [26] that, depending on the redox potential of the cell (this can be measured by the redox couple GSH/GSSG), alternatively promote different cellular responses that include proliferation, cell death by apoptosis, cell cycle arrest or other programs [63]. These considerations may provide a mechanistic linkage between the pro-oxidant and cytopathic effects of the virus described in the Vero E6 cell model (presented in Section 4).

However, similarly to Nrf2, NF-κB expression and activity were stimulated already in the earliest steps of the SARS-CoV-2 infection process, i.e., up to 6 hpi [13] (Figure 4). But, differently from Nrf2, NF-κB activation lasted in the more advanced steps of the viral replication cycle up to CPE execution, i.e., 24 hpi and onward. This may suggest a mechanism for the viral infection to evade the role of this transcription factor in the innate immunity mechanisms of the host cell that may intervene at virus entry into the cell and during viral replication, as well as in the adaptive response to the pro-oxidant and thiol-consuming effects of the virus.

In fact, this transcriptional protein is a redox-sensitive node in the regulatory interactome of the cell with a key role in the adaptive response to a number of stressors [13,26] and in the impaired immunity of the diseased host [15]. Therefore, SARS-CoV-2 infection appears to control different transcription factors in order to create optimal conditions for its replication and, in this context, NF-κB activation could be a main player in the development of its cytopathic and pro-inflammatory effects (Figure 2). Based on these assumptions, a role for this transcription factor in the inflammatory and degenerative complications of severe COVID-19 of elderly patients is more than conceivable, but remains to be conclusively demonstrated. The possibility that the pro-oxidant environment generated by the infection in the host cell [8] might sustain NF-κB activation as a response useful to promote viral replication, cannot be ruled out and deserves further investigation to shed more light on the pathobiology of SARS-CoV-2 infection.

These considerations and a series of in silico simulation studies [14,86], support the hypothesis that NF-κB and its cellular interactome may represent putative pharmacological targets of this infectious disease holding therapeutic potential in COVID-19.

## 7. Conclusions

This review article summarized the available evidence on the importance of cellular thiols in SARS-CoV-2 infection and in the development of severe COVID-19. It is now demonstrated that, similarly to other COVID and non-COVID viruses, SARS-CoV-2 may benefit from pro-oxidant conditions in both the extracellular and intracellular environment to complete its infectious cycle. In fact, as demonstrated in the case of other viral infections [73,74], a pro-oxidant environment might promote the virus-host cell interaction leading to increased affinity binding of the spike S protein to ACE 2 receptor. This binding could be influenced by the redox balance of LMW thiols in the extracellular fluids and of Cys residues on the extracellular domains of membrane proteins in the host cell [2]. In this respect, the available literature reviewed in this article indicates that the development of such a pro-oxidant environment in the lung and other tissues is clearly an age-dependent process. In fact, the redox balance of extracellular thiols and GSH levels of the lung, both linearly decline with age, and this may represent a major risk factor in the diseased host to become infected and to develop severe complications (Figure 3).

Furthermore, we recently described an active role for SARS-CoV-2 infection in inducing a defective metabolism and redox function of cellular GSH (Figure 2) [8], which is another important mechanism for this virus to promote pro-oxidant conditions in the host cell that are presumably useful to its replication. This effect was associated with an initial activation of Nrf2, which is followed by the induction of a hyporesponsive state of this transcription factor important in the adaptive homeostasis of the cell (Figure 4). This may reroute Cys utilization from GSH and cellular protein synthesis to the incorporation into viral proteins during viral replication. At the same time, these effects impair the adaptive homeostasis potential of the infected cell thus promoting cellular damage and the CPE of the virus by the induction of an increased flux of ROS and apoptotic signalling (Figure 2). ER stress is a main instigator of these processes in that it is the first and most important cellular abnormality induced by the viral replication, followed by the activation of stress and inflammatory kinases, and nuclear translocation of transcription factors as NF-κB and c-Jun (Figure 2). These are all redox-sensitive elements of the cellular signalling with key role in coordinating the stress response of the host cell with the control death and pro-inflammatory pathways [13]. Therefore, all these molecular and metabolic alterations induced by the SARS-CoV-2 infection that have been described for the first time at the cellular level in our laboratories in Vero E6 cells, indicate the existence of a complex redox reprogramming process that is part of the viral infection program and a leading cause of its CPE (Figure 4). The efficacy of antiviral agents in preventing the pro-oxidant and pro-inflammatory effects of the virus in the host cell has been demonstrated in these in vitro studies, indicating that an efficient inhibition of viral replication in the earliest phase of the viral cycle, i.e., before ER remodelling and stress activation [87], is an effective strategy to restore the cellular metabolism of GSH in the host cell. This type of effect was obtained using the viral protease inhibitor Nel, but not the nucleotide analogue Rem, thus indicating the importance of preventing the abnormal incorporation of cellular Cys in viral proteins as a way to preserve the availability of this amino acid for the synthesis and cytoprotective effects of GSH.

Antioxidants and Cys analogues, such as NAC, have been identified to further enhance the efficacy of Nel in restoring the cellular levels of GSH, which may suggest a strategy to recover the redox homeostasis of the infected tissue that is worth investigating in further pre-clinical and clinical studies.

In this article we also present the role of thiols in the defence against oxidative stress of tissues and especially of the lung, and since this function decreases with the subject’s age and with the presence of comorbidity [2], we postulated the possibility of providing a timely and precise diagnosis of a defective redox in the elderly. This may allow more efficient strategies of protection and prevention from severe COVID-19, and better clinical management of patients during both the acute phase of infection and recovery. In this respect, the Protein Thiolation Index has recently been described as a robust biomarker of the age-dependent decline of the thiol/disulphide balance in the extracellular fluids and of the corresponding decline of antioxidant and redox homeostasis systems of tissues [31,32]. We here propose that this parameter can be used, together with the determination of the reduced and oxidized forms of extracellular LMW thiols, to predict the risk of severe infection and clinical complications, as well as to monitor the effect of antiviral therapies and ancillary treatments with antioxidants such as thiol analogues that have already been used with some success to treat viral infections including those of the respiratory tract (reviewed in [60]).

In conclusion, the age-dependent decline of redox homeostasis and metabolic processes that control cellular thiols, and especially GSH, represent a risk factor for severe complications of SARS-CoV-2 infection, which is a pro-oxidant and thiol-depleting event itself. Further studies are needed to shed more light on the cellular mechanisms standing behind the pro-oxidant effects of viral replication to prevent them and recover the redox homeostasis of infected tissues.
